# Properties of CrSi_2_ Layers Obtained by Rapid Heat Treatment of Cr Film on Silicon

**DOI:** 10.3390/nano11071734

**Published:** 2021-06-30

**Authors:** Tatyana Kuznetsova, Vasilina Lapitskaya, Jaroslav Solovjov, Sergei Chizhik, Vladimir Pilipenko, Sergei Aizikovich

**Affiliations:** 1 Nanoprocesses and Technology Laboratory, A.V. Luikov Institute of Heat and Mass Transfer of National Academy of Science of Belarus, 15, P.Brovki str, 220072 Minsk, Belarus; t_kuzn@hmti.ac.by (T.K.); lapitskayava@hmti.ac.by (V.L.); chizhik_sa@tut.by (S.C.); 2 JSC “INTEGRAL”—“INTEGRAL” Holding Managing Company, 121 A Kazintsa, 220108 Minsk, Belarus; jsolovjov@integral.by (J.S.); office@bms.by (V.P.); 3 Research and Education Center “Materials”, Don State Technical University, 344000 Rostov-on-Don, Russia

**Keywords:** thin films, chromium, chromium disilicide, silicon substrate, rapid thermal treatment, roughness

## Abstract

The changes in the morphology and the electrophysical properties of the Cr/n-Si (111) structure depending on the rapid thermal treatment were considered in this study. The chromium films of about 30 nm thickness were deposited via magnetron sputtering. The rapid thermal treatment was performed by the irradiation of the substrate’s back side with the incoherent light flux of the quartz halogen lamps in nitrogen medium up to 200–550 °C. The surface morphology was investigated, including the grain size, the roughness parameters and the specific surface energy using atomic force microscopy. The resistivity value of the chromium films on silicon was determined by means of the four-probe method. It was established that at the temperatures of the rapid thermal treatment up to 350 °C one can observe re-crystallization of the chromium films with preservation of the fine grain morphology of the surface, accompanied by a reduction in the grain sizes, specific surface energy and the value of specific resistivity. At the temperatures of the rapid thermal treatment from 400 to 550 °C there originates the diffusion synthesis of the chromium disilicide CrSi_2_ with the wave-like surface morphology, followed by an increase in the grain sizes, roughness parameters, the specific surface energy and the specific resistivity value.

## 1. Introduction

The chromium films represent an important and multifunctional material in the various contemporary branches of industry owing to their exceptional properties (enhanced mechanical, corrosion, conductive, optical and catalytic properties) [1,2,3]. The chromium thin films (thick up to 200 nm) are widely applied as durable conductive coatings and contact pads in microelectronics and sensors [4,5]. Chromium can be applied as the underlayer material for the multipurpose coatings in the microelectronic devices and microsensor products on silicon and glass [6,7,8], where its morphology is important, as it determines the structure and properties of the subsequent functional layer [9]. In this case, chromium promotes the crystallization extent of the upper layer, increases the grain size and increases the Hall ratio and the concentration of carriers owing to the suppression of Na atom diffusion into the films (for glass) and reduces resistance. Meanwhile, the increase in roughness that takes place in the upper films with deposition on the underlayer of Cr enhances the functional parameters of the upper layer [6].

The electric conductivity of the chromium films, applied on the silicon substrate, substantially changes during the formation of the chromium disilicide (CrSi_2_) [10,11,12]. Such properties of CrSi_2_ as the high temperature of melting, the resistance to oxidation and the capability of standing a considerable deformation make it a prospective material under the conditions of the energy influences [11,13]. Earlier, CrSi_2_ layers were mainly applied as the barriers of Schottky diodes [3,4,5,11,14,15]. At present, the CrSi_2_ layers are also used as a joint between silicon and the working element in the integrated circuits and the sensors owing to the low transient resistance and the semiconductor properties [2,11]. This peculiarity in combination with a good compatibility with the regular silicon technologies makes it possible to successfully apply CrSi_2_ in the thermoelectric and photoelectric devices [1,2,16]. The narrow prohibited zone [17] makes it suitable for application in the converters and sensors in micro- and nanoelectronics. Meanwhile, the electrical properties of CrSi_2_ substantially depend on the method of formation and the microstructure.

The chromium disilicide layers may be formed by direct deposition of the given phase on the silicon substrate [12] and by means of modification of the chromium films at the expense of diffusion of silicon atoms into chromium [18]. This diffusion may progress even under low (<300 °C) temperatures [18]. The conventional heating (furnace annealing) is performed at the temperature of 450 °C and lasts for 30 min [16].

The standard temperature of transition of the nanocrystalline film of CrSi_2_ to the semiconductor state is the temperature of 1200 °C [19]. In the case of CrSi_2_ layers synthesized at 450 °C and possessing metallic conductivity, they can be subjected to annealing for 300 s. The progressive method of the rapid thermal treatment (RTT) of the silicon wafer with the applied metal layer makes it possible to obtain the required semiconductor layers at a substantially lower temperature, and the process parameters make it possible to control the electrical properties of the synthesized coatings [20]. An abrupt change in the electrical properties of the metallic films at the RTT temperature of 400 °C is related to the phase transitions [21]. The rapid thermal treatment of the silicon wafer with the applied Cr layer makes it possible to obtain the CrSi_2_ layers in several seconds and at a temperature of 400–600 °C [22].

The scientific literature presents no dependences relating the RTT temperature of the Cr films to their parameters of roughness, grain size, specific surface energies and resistivity. The purpose of the given paper is to study the influence of the RTT temperature of the chromium thin films, applied on the silicon substrate by means of the magnetron sputtering, on the parameters of the surface roughness, grain size, specific surface energy and resistivity.

## 2. Materials and Methods

### 2.1. Coating Deposition

The chromium films about 30 nm thick were applied on the silicon substrates by means of the magnetron sputtering of the chromium target with the purity of 99.5% in the argon medium with the purity of 99.993% under the Ar pressure of 0.35 Pa, at the Ar flow rate of 180 ccm, the power of discharge of 5.1 kW and the discharge current of 7.5 A (the power density constituted about 5.85 W/cm^2^ with the discharge voltage of 680 V) on the unit SNT (StratNanoTech) “Sigma” (StratNanoTech Invest, Minsk, Belarus). The silicon substrates were essentially the epitaxial layers of phosphorus-doped silicon with the resistivity of 0.58–0.63 Ω·cm and the thickness of 5.3–5.8 µm, formed on the substrates of p-type monocrystalline silicon with the resistivity of 10 Ω·cm and the orientation of (111).

Further on, the substrates were subjected to the rapid thermal treatment in the mode of the heat balance by means of irradiation of the reverse side of the substrates with the incoherent light flux of the quartz halogen lamps of the constant power in the nitrogen medium for 7 s to attain the temperature from 200 to 550 °C at atmospheric pressure on the unit JetFirst 100 (Jipelec Qualiflow, Montpellier, France). The heating rate was 30–80°/s. The temperature control of the working side of the substrate was performed by means of the thermal couple with the precision of ±0.5 °C.

### 2.2. Coating Characterization

The surface roughness parameters after the rapid thermal treatment of the Cr/Si structure were determined by means of AFM [23]. All studies of the surface morphology, roughness and adhesion forces (*F_ad_*) values were carried out using AFM Dimension FastScan (Bruker, Santa Barbara, CA, USA) in the PeakForce QNM (Quantitative Nanoscale Mechanical Mapping, Bruker, Santa Barbara, CA, USA ) mode with standard silicon cantilevers of the CSG10_SS type (TipsNano, Moscow, Russia) with a tip radius of 3.2 nm and console stiffness of 0.26 N/m. The probe performs an “approach–retraction” motion to the sample surface with the record of the forces curves at each point of the image. The forces between the AFM tip and the sample can be precisely controlled. Obtaining and recording entry of such curves is the basis of the PeakForce QNM mode, based on which recomputation of the values of the adhesion forces is automatically performed with consideration of the specific characteristics of the used probe. For each sample, 220–250 grains were considered to determine the grain size of Cr and CrSi_2_ films. The “Particle Analysis” function in the “NanoScope analysis” processing program was applied for this purpose.

The specific surface energy (*γ*) (the work of adhesion) was determined by means of the expression *γ =* *F_ad_/*(2π*R*) [24], where *F_ad_* is the force of adhesive interaction between the AFM probe tip and the surface, N, and *R* is the radius of the probe tip, m. *Ra*, *Rq* and *Rz* parameters were measured on 1 × 1 µm^2^ areas; the grain diameters were measured on 250 × 250 nm^2^ areas.

**Figure 1:**
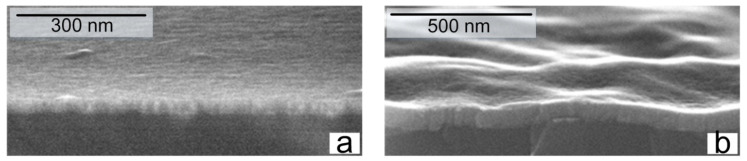
The initial Cr film (**a**) and CrSi_2_ film (**b**) obtained by RTT Cr at 400 °C.

The surface resistance (*Rs*) of the Cr/Si and CrSi_2_/Si layers was measured by means of the four-probe method with the unit RS-30 (KLA Tencor, Milpitas, CA, USA). The error of the surface resistance determination was less than 5%. Meanwhile, the thickness of the layers (*t*) was determined by means of the scanning electron microscope S-4800 (Hitachi, Tokyo, Japan). To measure the thickness of the films by the SEM (scanning electron microscopy ) method, the silicon wafer with the film was vertically cleaved. This cross-sectional cleavage was examined by SEM to determine the thickness (Figure 1). The resistivity value (*ρ*) was determined by means of the expression *ρ = Rs × t* [21].

## 3. Results and Discussion

**Figure 2:**
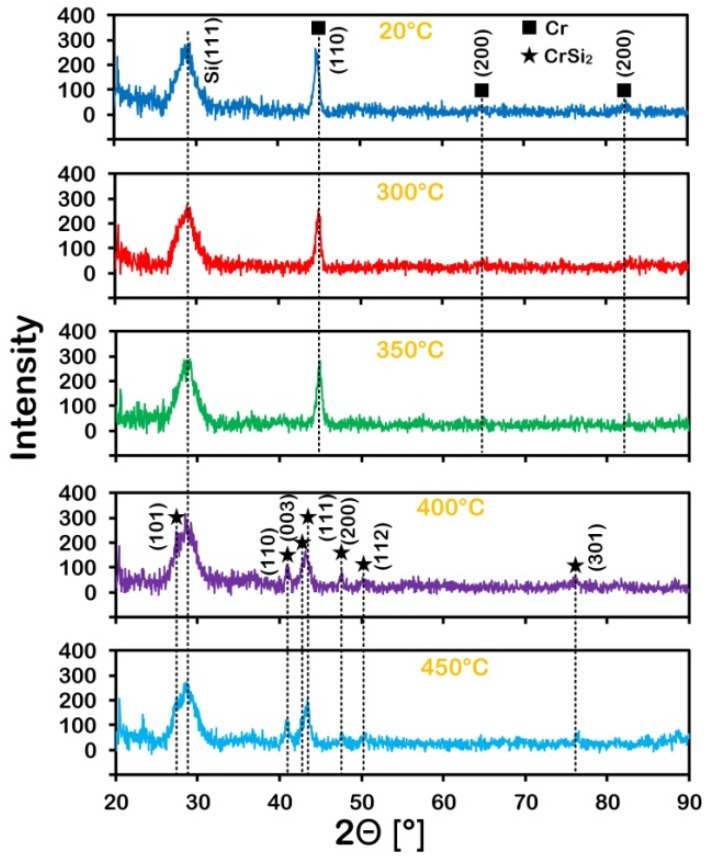
X-ray diffraction (XRD) patterns of Cr coatings in the initial state (20 °C) and after RTT with Cr lines (300 °C, 350 °C) and CrSi_2_ lines (400 °C, 450 °C).

Structural properties of the deposited Cr film and the Cr film after RTT measured by XRD are shown in Figure 2. The main phase in the initial film and after RTT at 300 °C and 350 °C is the Cr phase. The main phase after RTT at 400 °C and 450 °C is the CrSi_2_ phase.

**Figure 3:**
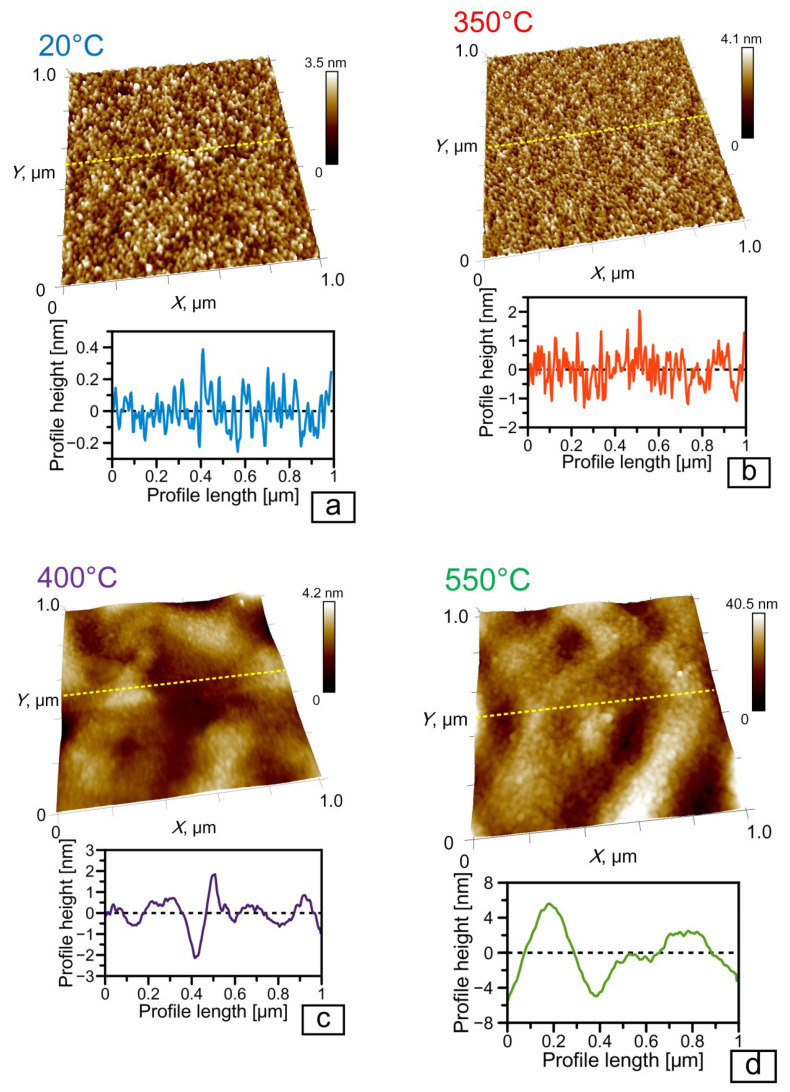
Morphology and the surface nanoprofiles of the Cr/Si structure after deposition and rapid thermal treatment: (**a**) initial film; (**b**) at the temperature of 350 °C; (**c**) at the temperature of 400 °C; (**d**) at the temperature of 550 °C.

The results of the investigations of the surface morphology of the obtained coating are represented in Figure 3. In the initial microstructure of Cr/Si, as well as after its rapid thermal treatment at the temperatures from 200 to 350 °C, one observes the smooth surface with the fine grain morphology (Figure 3a,b). At the RTT temperature of 400–550 °C, there arises the “wave-like” morphology of the Cr/Si structure surface (Figure 3c,d). The given changes are explained by the phase transition of the chromium film into the chromium silicide in the temperature range from 350 to 400 °C, which is well matched with the results of the earlier academic papers [10], where the phase transition of Cr to CrSi_2_ was observed at the temperature of 400 °C. The presence of the “wave-like” morphology in the structures of CrSi_2_/Si obtained by diffusion synthesis conforms well with the results of the literature [15,25] and is explained by the mismatch of the crystal lattice parameters of CrSi_2_ and Cr and the prevailing diffusion of silicon from the substrate to the boundary of Cr/Si. It is known that the parameter of the crystal lattice of CrSi_2_ by far exceeds the parameter of the crystal lattice of Cr (4.405 and 2.885 Å, respectively) [14,21]. Formation of CrSi_2_ starts at the boundary of Cr/Si under the layer of Cr and is realized by diffusion of Si from the substrate to the separation boundary and by embedding into the lattice of Cr with its subsequent transformation into CrSi_2_.

Table 1 shows the difference in the characteristics of the Cr films after the conventional heating and RTT of the same film at the same temperature. The complete absence of Cr lines in the XRD analysis of the Cr/Si structure after RTT treatment at 450 °C suggests that the top layer is CrSi_2_ (Figure 2). The thickness of the CrSi_2_ layer obtained by RTT is almost twice that after conventional heating. The difference in Cr thickness after conventional heating and RTT heat treatment is explained by the difference in the activation energy of the process. During RTT, additional stimulating factors act on the process of CrSi_2_ formation, reducing the activation energy of the process. Such factors can be the breaking of silicon–silicon bonds and the excitation of electrons in the silicon wafer under the action of photon flux.

At the time of the rapid thermal treatment (for several seconds) the entire layer of Cr is replaced with CrSi_2_, and as its volume is greater, distortion of the straight line of the boundary between the silicon wafer and the film occurs with distortion of the coating surface. Formation of CrSi_2_ in the system of Cr–Si is a most probable outcome on the assumption of the lowest standard enthalpy of formation (ΔH°) of an individual phase of CrSi_2_ amongst other probable phases [25]. The paper [26] indicates that chromium disilicide is formed at the expense of silicon atom diffusion and not because of chromium, as for metals a higher energy of activation is required, 1.4 times higher as a minimum.

**Figure 4:**
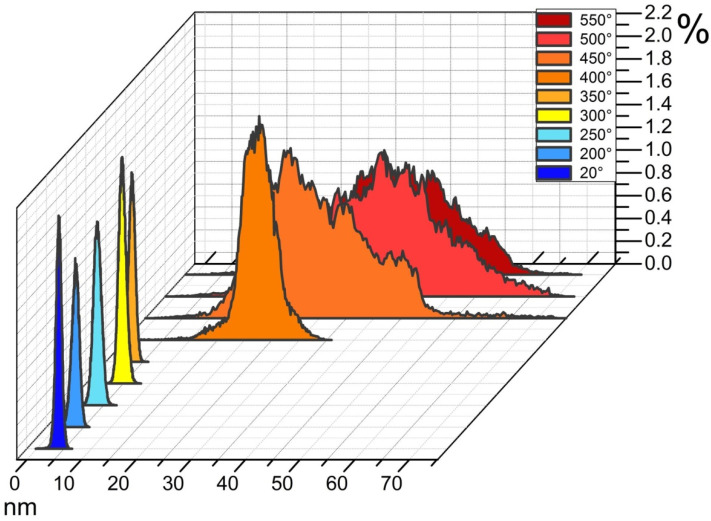
Histograms of the distribution densities of the height values of the nanoasperities on the surface of the structure with the size of 1 × 1 µm^2^ after deposition and rapid thermal treatment.

The surface morphological change during the phase transition of Cr → CrSi_2_ also determines the appropriate changes of its nanoprofiles (compare Figure 3a,b and Figure 3c,d). The comparative analysis of the samples’ nanoprofiles demonstrates an abrupt increase in both the average values of nanoasperities at the RTT temperature of 400 °C and of the incidences of their scatter (Figure 4). Thus, in the initial structure of Cr/Si and after its rapid thermal treatment at the temperature from 200 to 350 °C, the mean values of nanoasperities are within the limits from 2 to 7 nm. An increase in the RTT temperature from 400 to 550 °C results in the shift of the distribution maximum and in the scatter of the average values of nanoasperities from 10–35 nm to 30–60 nm appropriately. Such an evolution of the surface nanoprofiles is determined not only by the samples’ surface morphology transformation but also by the size change of the crystal grains.

**Figure 5:**
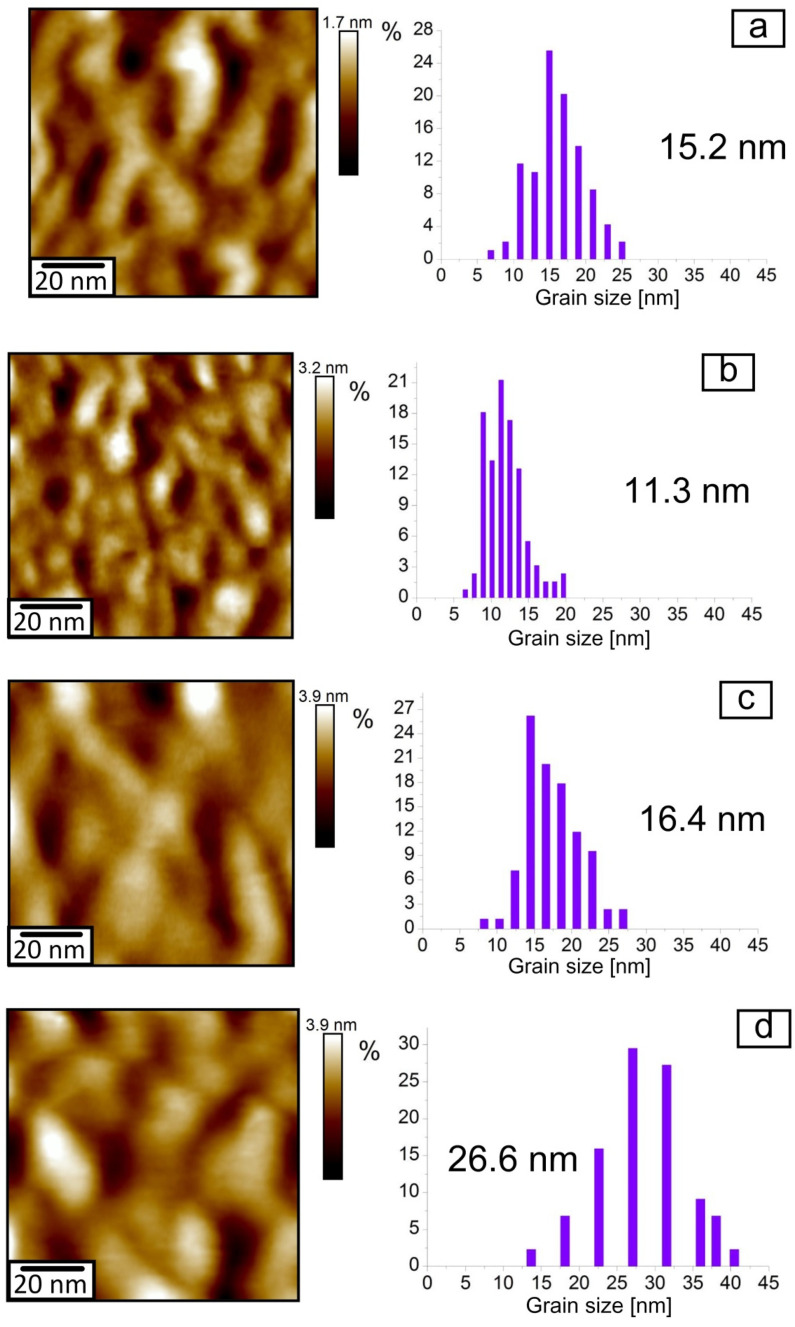
Atomic force microscope image (2D view) of the surface and the histograms of the distribution densities of the grain sizes of the Cr/Si structure after deposition and rapid thermal treatment: (**a**) initial film; (**b**) at the temperature of 350 °C; (**c**) at the temperature of 400 °C; (**d**) at the temperature of 550 °C.

Analysis of the changes in the grain sizes in the Cr/Si structure, obtained by means of AFM on the fields with the size of 100 × 100 nm^2^, with the increase in the RTT temperature confirms the given conclusion (Figure 5). The mean grain size in the initial film of Cr is about 15 nm (Figure 5a). At the RTT temperature of the Cr/Si structure from 200 to 350 °C, it is reduced to 10–12 nm (Figure 5b). This somewhat contradicts the classic presumptions in compliance with which the process of heating increases the grain size as during the prolonged time the collective recrystallization manages to occur [27]. However, in our case, this does not happen due to the high rate of heating and absence of the prolonged retention at the temperature.

Nonetheless, the given treatment results in recrystallization of the Cr films, accompanied by the density reduction of the structural and admixture defects, which enhances their density. At the RTT temperature of the Cr/Si structure from 400 to 550 °C, the average grain size increases from 16 to 26 nm, which is determined by the increase in the rate of the Si diffusion into the Cr film with the RTT temperature increase.

In all the applications of the thin films of Cr and CrSi_2_, the important parameters determining the level of their functional properties are the grain size and roughness of the surface. In some cases, the best option is low roughness, while in others, the best option is high roughness [6]. The values of the roughness parameters determined with the application of the atomic force microscope (AFM) can be found in academic papers on the electronic properties of the thin films of Cr and CrSi_2_ [1,2,5,6,28,29,30]. However, grain size investigation results are far more rarely found, although the atomic force microscope is a well-suited tool for the evaluation of the grain size in polycrystalline films [31,32]. For example, in [2], the grain size of the Cr film was close to that obtained in this work and was 13 and 14 nm at the negative bias voltage on the substrate of 50 and 250 V. At the negative bias on the substrate of 450 V, the grain size was 20 nm [2]. The roughness was also close and amounted to 0.57–1.49 nm.

**Figure 6:**
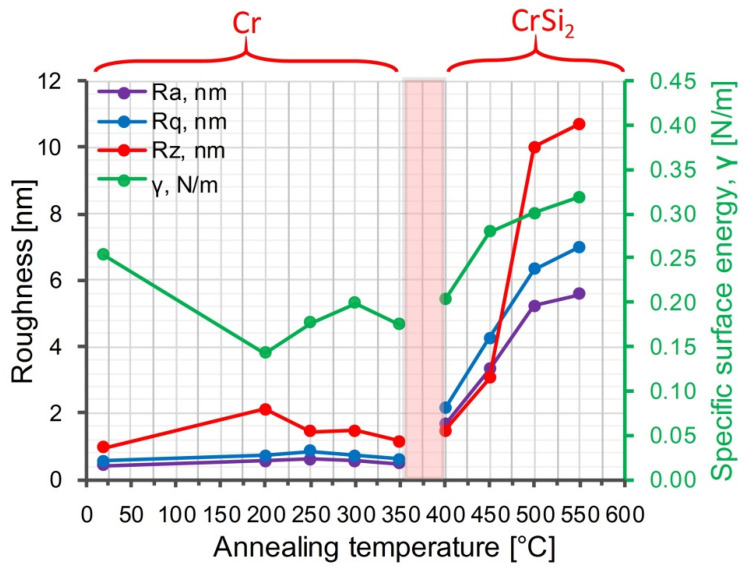
Dependences of the roughness parameters *Ra*, *Rq* and *Rz* and the specific surface energy of the Cr/Si structure on the rapid thermal treatment temperature.

Analyses of dependences the roughness parameters *Ra*, *Rq* and *Rz* on the RTT temperature (Figure 6) also show their relation, primarily, with the phase changes in the structure of Cr/Si. Roughness parameters *Ra* and *Rq* of the initial structure and after its rapid thermal treatment at the temperature from 200 to 350 °C are at the level of 0.5 nm and do not experience substantial changes. The *Rz* roughness parameter within the given RTT temperature range is at the level of 1.0–2.0 nm. The considerable changes in the values of the surface roughness parameters of the Cr/Si structure are observed in the result of the phase transition of Cr → CrSi_2_ with the RTT temperature increase from 400 to 550 °C. Thus, the *Ra* surface roughness parameter in the given RTT temperature range increases from 1.5 to 5.5 nm, the *Rq* parameter increases from 2.0 to 7.0 nm and the *Rz* parameter increases from 1.5 to 11.0 nm, reflecting the changes in the phase composition and the crystal structure.

The value of the specific surface energy in the samples with the initial structure of Cr/Si is at the level of 0.26 N/m and after the rapid thermal treatment at the temperature of 200 °C is reduced almost 2 times to values of the order of 0.14 N/m. The given behavior of the *γ* parameter serves as evidence of the unbalanced state of the initial Cr film, obtained immediately after the magnetron sputtering and containing the excessive mechanical stresses, structural defects and argon admixture atoms. Thus, the RTT of the Cr/Si structure results in the density reduction of the structural and admixture defects in the Cr film, as well as in the relaxation of the film’s mechanical stresses. The insignificant growth of values of the γ parameter up to 0.18–0.20 N/m with the RTT temperature increase to 250–350 °C is caused by the growth in the number of unbalanced states, related to the appropriate increase in the heating and cooling rate of the structure. The unbalanced states of some atoms are associated with the action of photon energy and additional breaking of Si–Si bonds. The changes of the *γ* parameter at the RTT temperatures from 400 to 550 °C, apparently, are determined both by the structural-phase transformations in the Cr/Si structure and the by the changing morphology of its surface, as well as by the cooling rate of the structure after the rapid thermal treatment.

**Figure 7:**
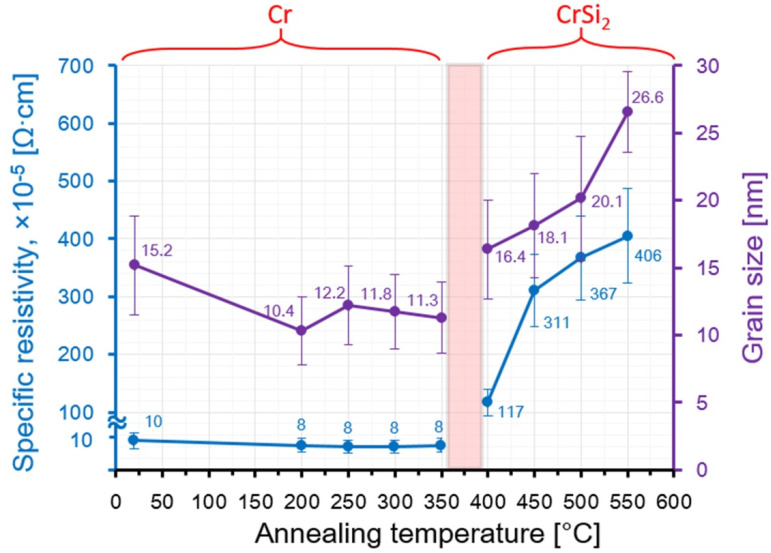
Dependences of resistivity and the mean grain size in the Cr film on the temperature of the rapid thermal treatment of the Cr/Si structure.

The specific resistivity value of the initial Cr thin films on silicon is about 1.0 × 10^−4^ Ω·cm, and after the rapid thermal treatment at the temperatures from 200 to 350 °C, it is reduced to 8.0 × 10^−5^ Ω·cm (Figure 5), which confirms the earlier assumptions about the recrystallization processes resulting in the density reduction of the admixture and structural defects and in the higher density of grains. The rapid thermal treatment at the temperature of 400 °C results in the avalanche growth of specific resistivity to values of the order of 1.2 × 10^−3^ Ω·cm, which is definitely determined by the transition of the Cr film to the CrSi_2_ phase. Within the RTT temperature range from 450 to 550 °C, one observes the growth in the specific resistivity from 3.1 × 10^−3^ to 4.0 × 10^−3^ Ω·cm. Comparison of the dependences of the specific resistivity and the grain sizes on the RTT temperature of the Cr/Si structure (Figure 7) makes it possible to draw a conclusion that the ρ value is directly influenced specifically by the grain size of the CrSi_2_ phase. The significant changes in all parameters (roughness, specific surface energy, grain size, thickness, surface resistivity) of the thin Cr and CrSi_2_ films are indicators of the phase transition in the films. The increased diffusion rate at 400–550 °C RTT is associated with the breaking of Si–Si bonds under the action of photon energy. The increased diffusion rate and the difference in the parameters of the crystal lattices of Cr and CrSi_2_ cause an increased surface roughness in the CrSi_2_ layers. A high correlation has been found between roughness and surface resistance.

## 4. Conclusions

By means of the atomic force microscope and the electrophysical measurements, the influence of the rapid thermal treatment temperature of the Cr/n-Si structure (111) with incoherent light flux irradiation at constant power on the back side of the silicon wafer for 7 s in the nitrogen medium on the surface morphology and the specific resistivity of the Cr films was studied.

It has been established that at the rapid thermal treatment temperature of the Cr/Si structure from 200 to 350 °C, the fine grain morphology of the initial Cr film is retained, as are the values of the surface roughness parameters *Ra*, *Rq* and *Rz*. Meanwhile, one observes an insignificant reduction in the grain size, specific surface energy and resistivity of the chromium films, which is determined by recrystallization of the chromium films, accompanied by the density reduction of the structural and admixture defects.

Within the rapid thermal treatment temperature range of the Cr/Si structure from 400 to 550 °C, the surface acquires the wave-like morphology determined by the phase transition of the Cr film to the CrSi_2_ layer with a greater parameter of the crystal lattice and is formed at the expense of silicon atom diffusion from the substrate to the layer in growth. The given phase changes are accompanied by the growth of the crystal grain size and determine the increase in the surface roughness parameters *Ra*, *Rq* and *Rz* and the specific surface energy and resistivity of the CrSi_2_ layers.

## Figures and Tables

**Table 1:** Comparison of the CrSi_2_ layer properties obtained by RTT and conventional heating.

Treatment	T (°C)	Time	Film Thickness (nm)	Ra (nm)	Rq (nm)	Rz (nm)	Grain Size (nm)	γ (N/m)	Specific Resistivity (10^−4^ Ω·cm)
RTT	450	7 s	60.0 *	3.33	4.25	3.1	18.1	0.28	31.1
Conventional heating		30 min	35.7	1.59	2.17	1.7	16.8	0.28	1.2

* The thickness of film after RTT; the thickness before RTT was about 30 nm.
